# Factors Associated With Frequent Exacerbation-Related Hospitalization in Chronic Obstructive Pulmonary Disease (COPD): A Hospital-Based Analytical Cross-Sectional Study From Central India

**DOI:** 10.7759/cureus.114622

**Published:** 2026-08-16

**Authors:** Shradhansh Agrawal, Manoj Kumar Prajapati, Kriti Dwivedi, Snehil Agrawal

**Affiliations:** 1 Pulmonary Medicine, Government Medical College, Satna, IND; 2 Internal Medicine, Government Medical College, Satna, IND; 3 Pulmonary Medicine, Shyam Shah Medical College, Rewa, IND; 4 Pathology, Faculty of Medicine and Health Sciences, Shree Guru Gobind Singh Tricentenary University, Gurugram, IND

**Keywords:** acute exacerbation, chronic obstructive pulmonary disease, frequent exacerbator, indoor air pollution, medication adherence, smoking

## Abstract

Background: Recurrent exacerbations of chronic obstructive pulmonary disease (COPD) are an important cause of hospitalization. The contribution of modifiable behavioral, environmental, and treatment-related factors may vary across clinical settings. This study evaluated factors associated with frequent exacerbation-related hospitalization among patients admitted with acute exacerbation of COPD in Central India.

Methods: This hospital-based analytical cross-sectional study enrolled 100 consenting adults with COPD who were admitted with an acute exacerbation between August 2, 2023, and August 3, 2024. Exposures were recorded at the index admission, and exacerbation-related hospitalizations during the preceding year were ascertained retrospectively using a study-specific prestructured proforma. The primary outcome was frequent exacerbation-related hospitalization, defined as two or more such hospitalizations during the preceding year. Categorical variables were compared using Pearson’s chi-square test or Fisher’s exact test, as appropriate, and unadjusted odds ratios (ORs) with 95% confidence intervals (CIs) were calculated.

Results: Sixty patients (60.0%) had two or more exacerbation-related hospitalizations during the preceding year. In unadjusted analyses, frequent exacerbation-related hospitalization was associated with older age (p = 0.024), smoking (OR 18.94, 95% CI 5.75-62.42), alcohol use (OR 2.67, 95% CI 1.15-6.21), indoor air pollution (OR 2.80, 95% CI 1.21-6.46), secondhand smoke exposure (OR 2.71, 95% CI 1.16-6.37), environmental dust exposure (OR 4.64, 95% CI 1.80-11.91), and medication discontinuation (OR 3.27, 95% CI 1.35-7.95).

Conclusions: Frequent exacerbation-related hospitalization was common and showed unadjusted associations with several potentially modifiable exposures and interruption of maintenance treatment. These exploratory findings support assessment of smoking, environmental exposures, and treatment continuity during admission but do not establish independent or causal effects.

## Introduction

Chronic obstructive pulmonary disease (COPD) is a major cause of chronic respiratory morbidity and premature mortality, with a disproportionate burden in low- and middle-income countries. Its clinical impact is amplified in settings where tobacco exposure, household air pollution, occupational dust, delayed diagnosis, and inconsistent access to maintenance treatment coexist [1,2]. For hospital-based respiratory services, an admission for acute exacerbation is therefore not only an episode requiring stabilization but also an opportunity to identify factors that may be contributing to recurrent disease instability.

COPD is diagnosed in the appropriate clinical context by persistent airflow obstruction on post-bronchodilator spirometry. An acute exacerbation is recognized clinically by a short-term worsening of dyspnea and/or cough and sputum, generally over fewer than 14 days [3,4]. Exacerbations severe enough to require emergency care or hospitalization are associated with impaired health status, recurrent healthcare use, and increased mortality [5-7].

A history of previous moderate or severe exacerbations is among the strongest predictors of future events [6]. The conventional frequent-exacerbator phenotype includes moderate exacerbations managed without admission as well as severe events requiring hospitalization; the present study examined only exacerbation-related hospitalizations during the preceding year [3,6]. Previous events alone, however, do not explain why some patients repeatedly require hospital admission. Older age, smoking, household or passive smoke exposure, and environmental or occupational dust have been associated with COPD morbidity and exacerbation risk. These factors often overlap in India and other resource-constrained settings [8-12].

Treatment continuity is another potentially modifiable factor. Interruption of prescribed maintenance therapy has been associated with symptom worsening and greater healthcare use. Conversely, inhaler education, adherence support, and structured respiratory follow-up may reduce preventable recurrence [13-16]. Identifying these factors during hospitalization can support practical interventions before discharge.

Hospital-based evidence from Central India remains limited regarding the factors associated with repeated exacerbation-related hospitalization. This study therefore evaluated demographic, behavioral, environmental, and treatment-related factors associated with frequent exacerbation-related hospitalization among patients admitted with acute exacerbation of COPD.

## Materials and methods

Study design and setting

This hospital-based analytical cross-sectional study assessed each participant once during the index admission and retrospectively ascertained exacerbation-related hospitalizations during the preceding year. It was conducted in the Department of Respiratory Medicine, R.D. Gardi Medical College and C.R. Gardi Hospital, Ujjain, Madhya Pradesh, India, from August 2, 2023, to August 3, 2024.

Study population and sample size

Adults with an established clinical diagnosis of COPD recorded by the treating respiratory team who were admitted with an acute exacerbation and provided written informed consent were eligible. An acute exacerbation was defined clinically as worsening dyspnea and/or cough and sputum over fewer than 14 days [3,4].

A preliminary pilot study conducted among comparable patients admitted with acute exacerbation of COPD found that approximately 66% had experienced two or more exacerbation-related hospitalizations during the preceding year. Using this proportion (p=0.66), a 95% confidence level, and an absolute precision of 10%, the minimum sample size calculated using the formula n=Z²p(1−p)/d² was 87. After allowing for 10% anticipated non-participation, the required sample size was 97. A total of 100 participants were ultimately enrolled.

Data collection and study variables

A study-specific pre-structured proforma was used during the index admission. Smoking was recorded as a binary history together with duration and pack-years, and alcohol use was recorded as a binary history together with duration and amount. Indoor air pollution referred to reported exposure to coal- or wood-burning stoves, secondhand smoke to passive smoke exposure, and environmental dust to reported chemical fumes or dust in the residential or occupational environment. Medication discontinuation was defined as a patient-reported history of having stopped prescribed maintenance COPD medication; its timing, duration, reason, access barriers, and inhaler technique were not separately categorized.

Outcome definition

The primary outcome was frequent exacerbation-related hospitalization, defined as two or more exacerbation-related hospitalizations during the preceding year. Patients with fewer than two such hospitalizations formed the comparison group. Moderate exacerbations managed without hospitalization were not captured.

Statistical analysis

Continuous variables were summarized as mean and standard deviation or as median and range, as appropriate. Categorical variables were summarized as numbers and percentages. Associations between categorical variables and hospitalization-frequency category were assessed using Pearson’s chi-square test. Fisher’s exact test was used when expected cell counts were small. Unadjusted ORs with 95% CIs were calculated for frequent exacerbation-related hospitalization. No multivariable adjustment or correction for multiple comparisons was performed; therefore, the analyses were considered exploratory. Statistical analyses were performed using IBM SPSS Statistics for Windows, Version 21 (Released 2012; IBM Corp., Armonk, New York, United States).

Ethical considerations

The study protocol was approved by the Institutional Ethics Committee, R.D. Gardi Medical College, Ujjain (IEC-RDGM; approval/reference number: PG/RESPIRATORY MEDICINE/83; date of approval: August 2, 2023). Written informed consent was obtained from every participant before enrollment.

## Results

A total of 100 patients hospitalized with acute exacerbation of COPD were included. The mean age was 62.9 ± 8.1 years. Forty patients had fewer than two exacerbation-related hospitalizations, whereas 60 had two or more and were classified as having frequent exacerbation-related hospitalization. Baseline demographic characteristics are presented in Table 1.

**Table 1:** Baseline demographic characteristics of the study population (N = 100).

Characteristic	Category	n (%)
Age group	≤50 years	17 (17.0)
Age group	51-60 years	26 (26.0)
Age group	61-70 years	44 (44.0)
Age group	>70 years	13 (13.0)
Sex	Male	93 (93.0)
Sex	Female	7 (7.0)
Marital status	Married	96 (96.0)
Marital status	Unmarried	4 (4.0)
Education	No formal schooling	55 (55.0)
Education	Primary school	44 (44.0)
Education	High school/college	1 (1.0)
Occupation	Farmer	67 (67.0)
Occupation	Non-farming occupation	33 (33.0)
Residence	Rural	88 (88.0)
Residence	Urban	12 (12.0)

**Figure 1:**
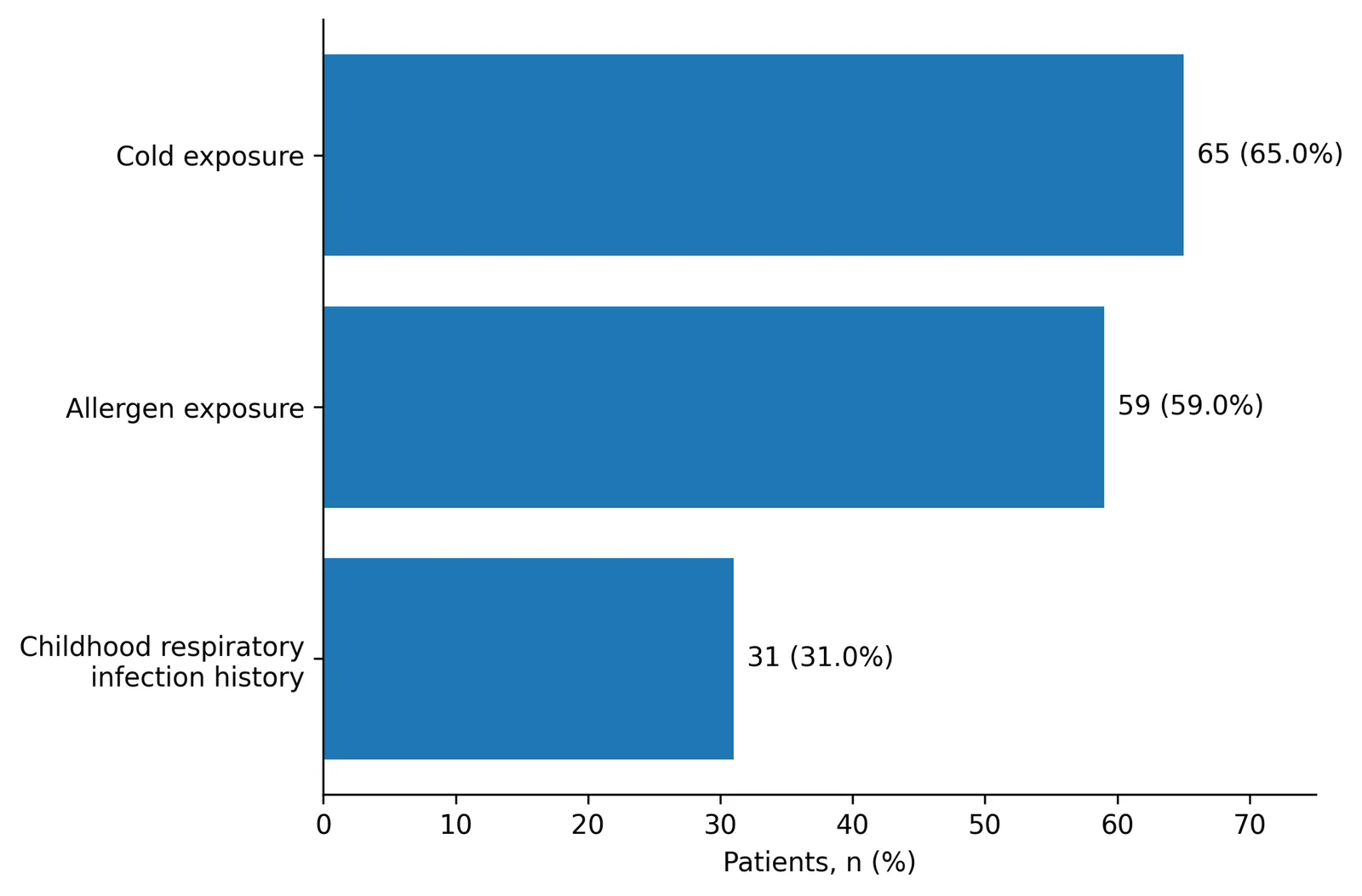
Additional recorded exposure history among hospitalized patients with COPD. COPD: Chronic obstructive pulmonary disease

Frequent exacerbation-related hospitalization was observed in 60 patients (60.0%). Cold exposure was reported by 65 patients (65.0%), allergen exposure by 59 (59.0%), and childhood respiratory infection by 31 (31.0%) (Figure 1).

Factors associated with frequent exacerbation-related hospitalization

Age group was associated with frequent exacerbation-related hospitalization (p = 0.024). The proportion of frequent exacerbators increased from five of 17 patients (29.4%) among those aged 50 years or younger to 30 of 44 patients (68.2%) among those aged 61-70 years and 10 of 13 patients (76.9%) among those older than 70 years. Demographic and behavioral associations are shown in Table 2.

**Table 2:** Demographic and behavioral factors associated with frequent exacerbation-related hospitalization. Values are presented as column percentages within each hospitalization-frequency group. The Pearson chi-square test was used to compare categorical variables between the two groups. Odds ratios represent the odds of having ≥2 exacerbation-related hospitalizations compared with the reference category. OR: odds ratio; CI: confidence interval; df: degrees of freedom. #Non-farming occupations comprised daily-wage workers (n=14), shop owners (n=9), homemakers (n=3), tailors (n=2), unemployed participants (n=2), and one participant each working as a laborer, peon, and stone-crusher worker. These categories were combined for association analysis because of sparse individual cell counts

Factor	Category	<2 hospitalizations, n (%)	≥2 hospitalizations, n (%)	Unadjusted OR (95% CI)	χ² statistic (df)	p-value
Age group	≤50 years	12 (30.0)	5 (8.3)	Reference	9.464 (3)	0.024
	51-60 years	11 (27.5)	15 (25.0)	3.27 (0.89-12.03)		
	61-70 years	14 (35.0)	30 (50.0)	5.14 (1.52-17.44)		
	>70 years	3 (7.5)	10 (16.7)	8.00 (1.52-42.04)		
Sex	Female	3 (7.5)	4 (6.7)	Reference	0.026 (1)	0.873
	Male	37 (92.5)	56 (93.3)	1.14 (0.24-5.37)		
Occupation#	Non-farming occupation	17 (42.5)	16 (26.7)	Reference	2.721 (1)	0.099
	Farmer	23 (57.5)	44 (73.3)	2.03 (0.87-4.75)		
Smoking	No	23 (57.5)	4 (6.7)	Reference	31.465 (1)	<0.001
	Yes	17 (42.5)	56 (93.3)	18.94 (5.75-62.42)		
Alcohol use	No	28 (70.0)	28 (46.7)	Reference	5.303 (1)	0.021
	Yes	12 (30.0)	32 (53.3)	2.67 (1.15-6.21)		
Tobacco chewing	No	24 (60.0)	47 (78.3)	Reference	3.918 (1)	0.048
	Yes	16 (40.0)	13 (21.7)	0.41 (0.17-1.00)		

Smoking showed the strongest association. Among smokers, 56 of 73 patients (76.7%) were frequent exacerbators, compared with four of 27 non-smokers (14.8%). Alcohol use was also associated with frequent exacerbation-related hospitalization. Tobacco chewing showed an inverse association at the threshold of statistical significance. Sex and occupation were not significantly associated with exacerbation-frequency category.

Environmental and treatment-related associations are presented in Table 3. Frequent exacerbation-related hospitalization was more common among patients with indoor air pollution, secondhand smoke exposure, and environmental dust exposure. Medication discontinuation was reported by 48 of 60 frequent exacerbators (80.0%) and 22 of 40 non-frequent exacerbators (55.0%). A history of pneumothorax was not significantly associated with exacerbation-frequency category.

**Table 3:** Environmental and clinical-history factors associated with frequent exacerbation-related hospitalization Values are presented as column percentages within each hospitalization-frequency group. Pearson chi-square test was used to compare categorical variables between the two groups. Odds ratios represent the odds of having ≥2 exacerbation-related hospitalizations compared with the reference category. OR: odds ratio; CI: confidence interval; df: degrees of freedom.

Factor	Category	<2 hospitalizations, n (%)	≥2 hospitalizations, n (%)	Unadjusted OR (95% CI)	χ² statistic (df)	p-value
Indoor air pollution	No	21 (52.5)	17 (28.3)	Reference	5.949 (1)	0.015
	Yes	19 (47.5)	43 (71.7)	2.80 (1.21-6.46)		
Secondhand smoke	No	19 (47.5)	15 (25.0)	Reference	5.414 (1)	0.020
	Yes	21 (52.5)	45 (75.0)	2.71 (1.16-6.37)		
Environmental dust	No	18 (45.0)	9 (15.0)	Reference	10.959 (1)	<0.001
	Yes	22 (55.0)	51 (85.0)	4.64 (1.80-11.91)		
Pneumothorax	No	37 (92.5)	53 (88.3)	Reference	0.463 (1)	0.496
	Yes	3 (7.5)	7 (11.7)	1.63 (0.40-6.71)		
Medication discontinuation	No	18 (45.0)	12 (20.0)	Reference	7.143 (1)	0.008
	Yes	22 (55.0)	48 (80.0)	3.27 (1.35-7.95)		

## Discussion

In this hospital-based study from Central India, frequent exacerbation-related hospitalization was common. The principal associations (unadjusted) involved older age, smoking, alcohol use, indoor air pollution, secondhand smoke exposure, environmental dust exposure, and medication discontinuation. The pattern is consistent with the clinical importance of recurrent exacerbations as a recognizable COPD phenotype and with the need to evaluate risk factors whenever a patient is hospitalized [5,6].

The high proportion of patients with frequent exacerbation-related hospitalization should be interpreted in the context of inpatient recruitment, which preferentially captures patients with severe or recurrent events. Even so, the finding has practical relevance. A patient who has required repeated hospitalization represents a high-risk contact in whom acute treatment should be accompanied by a review of potentially modifiable contributors.

Older age was associated with frequent exacerbation-related hospitalization. This may reflect longer disease duration, cumulative exposure to tobacco and household or occupational pollutants, age-related physiological vulnerability, and a greater burden of coexisting illness. Longitudinal evidence also indicates that lung-function trajectories across adult life influence the later expression and clinical risk of COPD [12].

Smoking had the strongest observed association. Tobacco smoke is central to COPD development and progression and may promote airway inflammation, mucus production, susceptibility to infection, and symptom instability [3,8]. The magnitude of the unadjusted association in this cohort supports routine assessment of smoking status and delivery of cessation counseling during every admission for acute exacerbation.

Environmental exposures were another prominent signal. Indoor air pollution, secondhand smoke, and environmental dust were each associated with frequent exacerbation-related hospitalization. These exposures may overlap with one another and with rural residence, farming, and smoking. Because no multivariable adjustment was performed, potential collinearity and residual confounding may partly account for the observed associations, and the independent contribution of each exposure cannot be determined. This finding is especially relevant to rural and semi-rural populations, in whom biomass fuel, passive smoke, agricultural dust, workplace particulates, and ambient pollution may coexist. Evidence concerning never-smoker COPD, wood-smoke exposure, occupational exposure, and short-term air pollution supports the biological plausibility of these associations [8-11].

Alcohol use was also associated with frequent exacerbation-related hospitalization, although the study did not quantify alcohol exposure or adjust for its correlation with smoking and other factors. Tobacco chewing showed an inverse association at the statistical threshold; this unexpected finding should not be interpreted as protective because the analysis was unadjusted and the exposure was based on history.

Medication discontinuation was strongly associated with frequent exacerbation-related hospitalization and represents a directly actionable factor. Previous studies have linked poor adherence to maintenance treatment with exacerbation occurrence and greater healthcare use. Reported barriers include cost, incorrect inhaler technique, insufficient education, comorbid illness, and inadequate follow-up [13-15]. Medication reconciliation, inhaler-technique review, and individualized adherence support should therefore be incorporated into discharge planning.

The absence of a statistically significant association with sex should be interpreted cautiously because the cohort was predominantly male, limiting evaluation of sex-related differences. Likewise, the broad classification of occupation as farming or non-farming may have obscured differences in the type, intensity, and duration of occupational exposure.

These findings support a brief determinant-focused assessment at admission or before discharge. Patients with recurrent hospitalization may benefit from smoking-cessation support, advice on household and passive smoke reduction, occupational protection, inhaler education, medication-adherence interventions, and early respiratory follow-up. Pulmonologist follow-up after a COPD admission has been associated with a lower risk of readmission [16].

Limitations of the study

This single-center analytical cross-sectional study included a small, predominantly male inpatient sample, limiting generalizability. Exclusion of patients whose acute respiratory distress precluded consent or study assessments may have underrepresented the most severely ill patients. Spirometric reconfirmation during the index admission was unavailable for 55 participants, creating potential diagnostic misclassification unless prior spirometric confirmation can be documented. Several exposures and previous hospitalization history were based on participant recall and were recorded using broad binary categories, creating possible recall and exposure or outcome misclassification. The farming/non-farming classification may also have obscured occupational heterogeneity. COPD severity variables were not included in the present association analysis. Multiple bivariate comparisons were performed without correction for multiplicity. Most importantly, the analyses were unadjusted; correlated exposures, collinearity, and residual confounding cannot be excluded, and the ORs should be interpreted as exploratory associations rather than independent or causal effects.

## Conclusions

In this single-center inpatient cohort, frequent exacerbation-related hospitalization was common and showed unadjusted associations with older age, smoking, alcohol use, indoor air pollution, secondhand smoke exposure, environmental dust exposure, and medication discontinuation. These exploratory findings do not establish independent or causal effects. They nevertheless identify clinically relevant factors that may be assessed during admission and addressed through smoking-cessation support, exposure-reduction advice, adherence counselling, and post-discharge follow-up. Larger multicenter studies with adjusted analyses are needed to clarify independent associations.
